# 3D Printing of TPU‐Liquid Metal Composite Inks for the Preparation of Flexible Sensing Electronics

**DOI:** 10.1002/open.202300301

**Published:** 2024-04-26

**Authors:** Shuting Liang, Mengjun Huang, Dabo Jiang, Jianyang Chen, Liang Hu, Jiujia Chen, Zhezi Wang

**Affiliations:** ^1^ College of Chemical and Environmental Engineering Chongqing Key Laboratory for Resource Utilization of Heavy Metal Wastewater Chongqing University of Arts and Sciences Chongqing 402160 PR China; ^2^ Key Laboratory of Intelligent Textile and Flexible Interconnection of Zhejiang Province Institution Hangzhou 310018 China; ^3^ Beijing Advanced Innovation Center for Biomedical Engineering School of Biological Science and Medical Engineering Beihang University Beijing 100191 China

**Keywords:** Liquid metal, Polyurethane elastomer rubber (TPU), Flexible composite fiber, 3D printing, Sensing function

## Abstract

Direct 3D printing of liquid metal is difficult to form and easy to destroy. In this paper, we developed a 3D printed composite material consisting of a thermoplastic polyurethane (TPU) matrix and liquid metal (LM) dispersed droplets, and introduced the method for realizing 3D printed devices with this composite material: First, the LM is added to 10~50wt %TPU at 190~200 °C through ultrasonic blending to prepare blended ink. After solid cooling, the LM‐TPU composite fiber with a diameter of 600 μm was prepared by Wellzoom desktop extruder at 190 °C at an extrusion speed of 400 mm/min. It has excellent elasticity, with a tensile limit of 0.637 N/m^2^, and the TPU could evenly wrap LM droplets. Finally, the LM‐TPU fiber is 3D printed at 240 °C by using a 3D printer, and 2D/3D flexible electronic devices with heating and conductive functions could be prepared. The microcircuit has good electrical conductivity; after adding voltage, the circuit has heat release; it could be used as heating equipment to keep warm and used in various flexible wearable electronic products.

## Graphical abstract

A composite ink composed of polyurethane matrix and liquid metal dispersion drops was developed, and 3D printing of the composite ink was successfully realized.

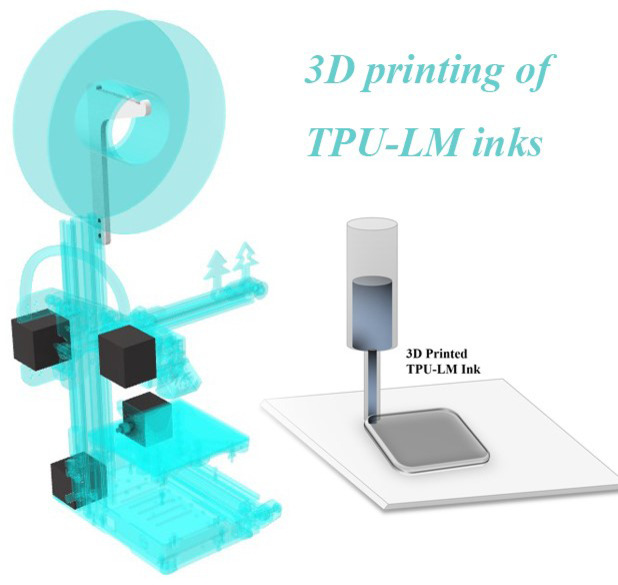

## Introduction

Three‐dimensional (3D) printing is called additive manufacturing, which could be processed by stacking materials layer by layer, saving materials and processing time. It is based on digital model files, and uses sticky materials such as powdered metal or plastic to construct objects by printing them layer by layer.[^1^, ^2^, ^3^] 3D printing technology could manufacture complex 3D functional structure sensors, and could complete the flexible sensor manufacturing of various sizes and complex shapes at one time, without the need for related mold manufacturing, saving costs. 3D printing products could be widely used in consumer electronics, medical, military, aerospace, and other art fields.

3D printing could choose different materials to use; the commonly used materials are divided into metal and non‐metal. Non‐metallic materials include nylon glass, polylactic acid, ABS resin, gypsum, etc. Metal materials include aluminum alloy, titanium alloy, stainless steel, copper powder, silver, alloy powder, etc. However, they have the disadvantages of complicated preparation processes, complicated chemical reactions, and very high curing temperatures. Conventional electronic printing inks mainly include nano‐gold, silver, copper, carbon nanotubes, graphene and so on. There are some shortcomings such as complex preparation process, low conductivity, complicated chemical reaction for wire formation and high curing temperature.

Traditional 3D printing of non‐metallic materials mainly includes printing various plastics, polymers, etc., but the printed objects are not conductive and could not prepare functional electronic devices. After 3D printing of printing consumables such as TPU and PLA on the market, the printed devices often cannot conduct electricity and cannot achieve specific functional effects.

Liquid metal composites are an ideal material for 3D printing.[^4^, ^5^, ^6^] Due to the surface tension of the LM, the liquid state at room temperature, when printing pure LM, it is difficult to directly 3D print into a solid conductive circuit. Therefore, by breaking down the LM into various micron and nanodroplets,^[7]^ polymers are added for printing.[^8^, ^9^] Compared with the previous ink, the LM composite ink has the characteristics of direct printing, high conductivity, wide application, moderate cost, and convenient preparation method.[^10^, ^11^, ^12^, ^13^, ^14^, ^15^]

In the composite ink formed by LM, the matrix is polymer, and the LM particles are filler.[^16^, ^17^, ^18^, ^19^, ^20^, ^21^, ^22^] For instance, liquid metal and polyurethane sponge (PUS), polymethacrylate (PMA), polydimethylsiloxane (PDMS), silicone resin, silicone rubber, polyester sponge, styrene‐butadiene‐styrene rubber (SBS), etc., could be used to form LM‐polymer composites with different properties. By pumping the liquid metal into the porous polyurethane sponge, the PUS‐LM composite ink could be prepared, and the prepared solid material has a high tensile strength of 1.11 MPa and a high conductivity of 1.87×10^6^.^[23]^ A PDMS/PUS/LM composite with high tensile strength (1.11 MPa) and conductivity (10^4^ S/cm) could be obtained by using a vacuum permeation method to permeate LM into a porous sponge PUS and packaging with polydimethylsiloxane.^[24]^ Using LM mixed with uncured liquid silicon or polyurethane to form materials with unique conductive elastic properties, the dielectric constant could be increased to 400 %.[^25^, ^26^] The mixture of polydimethylsiloxane liquid alloy, silicone resin‐based liquid alloy, polyester sponge, and LM gives the composite excellent elasticity and electrical conductivity.^[27]^

According to existing literature reports, LM‐embedded elastomer (LMEE) ink could be 3D printed, and three‐dimensional shapes could be created using this direct ink writing method.[^28^, ^29^] After a comprehensive summary of the patterning methods of LM soft electronic products, we could understand various patterns of LM patterning methods.^[30]^ With the development of LM fragment eutectic GaIn alloy and silver nanowire skeleton electrodes, the fabrication of complex stretchable electronic devices was now accessible.^[31]^

Currently, in the preparation of composite materials by LM‐polymer,[^32^, ^33^, ^34^, ^35^, ^36^, ^37^, ^38^] there are few reports on the formation of composite materials by LM and TPU and their use in 3D‐printed devices. TPU is a thermoplastic polyurethane elastomer rubber with excellent properties of high tension, toughness, and aging resistance. It is a mature and environmentally friendly material. By changing the proportion of each reaction component of TPU, products with different hardness could be obtained. With the increase of hardness, the products still maintain good elasticity and wear resistance. TPU products have excellent load‐bearing capacity, impact resistance, shock absorption properties, and high mechanical strength. TPU glass transition temperature is low, good elasticity at −35 °C, excellent cold resistance, flexibility, and other physical properties. It could be processed using standard thermoplastic material processing methods, such as injection molding, extrusion, calendaring, etc. It has good processability and could be processed with some polymers to obtain alloys with complementary properties.

In this paper, conductive liquid metal and TPU composite ink are developed, and different proportions of TPU and liquid metal are combined. By mixing LM with TPU polymer material, new additional functions are given to it through chemical and physical means, so that it becomes a functional fiber with physical properties, and its corresponding electrical and thermal properties are studied. The electrical conductivity and heat dissipation of the TPU‐LM fiber were studied. Using the produced fibers as 3D printing raw materials, based on liquid metal inks with excellent gold properties, flexible electronic products have been manufactured through printing, including 3D circuits and wearable health monitoring devices. The use of newly developed liquid metal inks in electronic skin and wearable devices through 3D printing to print highly stretchable circuits and achieve commercialization.

## Results and Discussion

### Preparation of TPU‐Liquid Metal Ink

**Figure 1:**
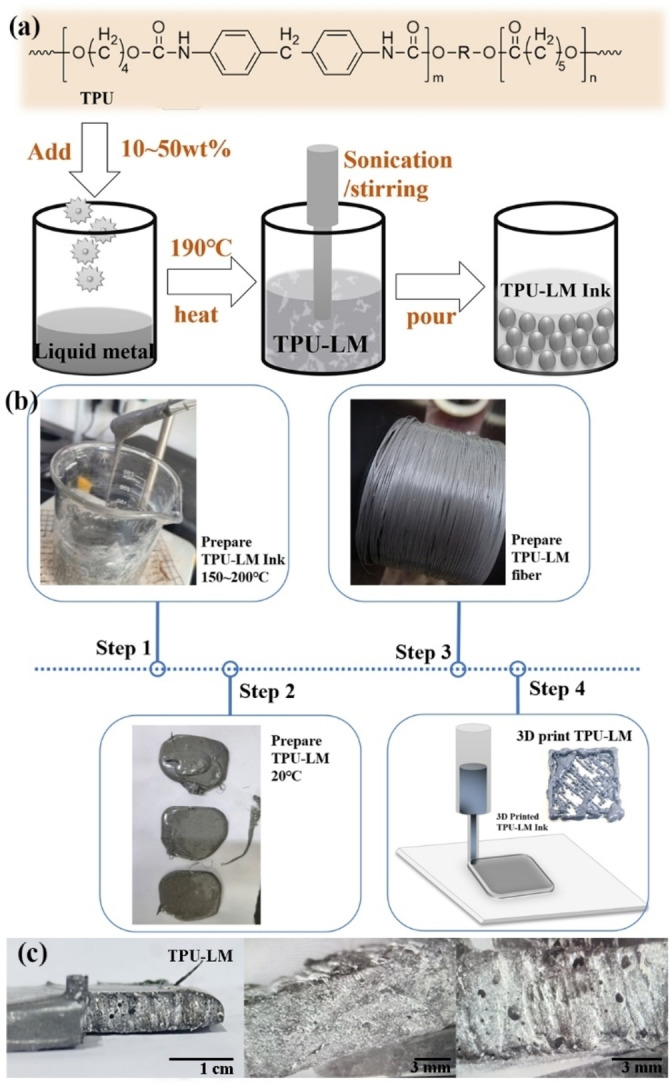
(a) Preparation method of TPU‐LM composite ink; (b) The entire process of from TPU‐LM inks to composite materials, the preparation of fibers and 3D printing TPU‐LM ink; (c) Cross‐section of TPU‐LM composites.

TPU (([CONHRNHCOOR′O]‐[CONHRNHCOO~O]_m_)_n_) is used as the substrate of polymer material, which is mixed with a liquid metal to form the original fiber material. TPU is a polymer material formed by reaction polymerization of diisocyanate molecules such as diphenylmethane diisocyanate (MDI) or toluene diisocyanate (TDI) with macromolecular polyols and low molecular polyols (chain extender). Because its surface tension and firm fluidity limit LM, pure LM inks often gather into spheres with low surface energy when they are directly 3D printed into patterns, and it isn′t easy to form the required continuous pattern. After the polyurethane coating is added to the LM, the bulk LM could be decomposed into micron ink particles in the polyurethane coating, which reduces the influence of enormous surface tension on the patterning of the LM. At the same time, polyurethane itself is also an excellent 3D printable material. Adding polyurethane would be conducive to further good printing of composite inks. The molecular formula of TPU is shown in Figure 1a.

The preparation method of TPU‐LM composite ink material is as follows: The prepared 107.4 g liquid metal is heated to 190 °C, then 40.4 g of TPU solid material cut into small particles is added. The composite material is put into a beaker and stirred at high temperatures, as shown in Figure 1a. The TPU‐LM blended ink was obtained after uniform mixing, as shown in Figure 1c. The liquid metal droplet is shattered by high‐intensity ultrasonic waves to obtain liquid metal droplet dispersion ink. The ultrasonic microwave method could break the liquid metal into micro/nanodroplets and disperse them in the polymer polyurethane solution to form stable micron LM droplets; the outer coating is polyurethane. This ink is gray in appearance, could flow at high temperatures, and is solid at room temperature, as shown in Figure 1d. This ink composition ratio is 38 wt %TPU+62 wt% LM material. This ink is composed of TPU polymer material covering liquid metal micro‐nano particles, in which TPU polymer material mainly covers liquid metal small particles.

The liquid metal and small particle TPU were mixed in the beaker, and 50 wt%, 38 wt%, and 10 wt% TPU were mixed with the liquid metal. They are heated at 190 °C to melt and constantly stirred to form a mixed uniform viscous ink, with a certain fluidity. The mixture is removed while hot and cooled to obtain solid TPU‐LM composites (Figure 1d). The mixture is broken into small particles (Figure 1e). The obtained small particles are added to the fiber extruder and slowly and evenly extruded at 190 °C to form micron TPU‐LM functional fibers.

The melting point of TPU polymer material is 190–230 °C, while liquid metal is liquid at room temperature. TPU polymer materials are very good at melting and mixing with liquid metals to form composite inks. The melting point of TPU 38 wt%‐LM composite ink was tested, and it was found that the melting point was 149.8 °C, and liquid flow TPU‐LM ink was obtained. Pour the fused blended ink into the square mold, let it stand for 30 minutes, cool down to room temperature, and get solid TPU‐LM 3D printing consumables, as shown in Figure 1ef. The cross‐section of TPU‐LM composite material was photographed under an electron microscope, and the cross‐section had a small number of porous structures and particle reflections, as shown in Figure 1h.

TPU‐LM ink has excellent printability. The 3D printing in TPU‐LM ink could be divided into three stages:

1. The initial state of TPU‐LM ink (100~200 °C) is liquid ink, and it has a certain fluidity;
2. It is cooled to room temperature to form a TPU‐LM composite sheet material, which could be extruded from the nozzle of a desktop 3D printing consumables machine;
3. The TPU‐LM long fiber roll is placed in the feed mouth of the 3D printer, and the material is heated to 170~200 °C by the probe, and then different shapes could be printed.

LM‐TPU composite ink has a firm adhesion to the glass substrate, and when it is attached to the glass, it can be scraped off with a knife. When the substrate is PET material, the solid material formed after the solidification of LM‐TPU ink is more accessible to separate from the PET substrate. When the ink viscosity is too high, it may block the nozzle of the 3D printer equipment, causing equipment loss and increasing maintenance costs. When the ink viscosity is exceptionally high, 3D printing becomes more difficult.

### Performance Test of TPU‐LM Ink

**Figure 2:**
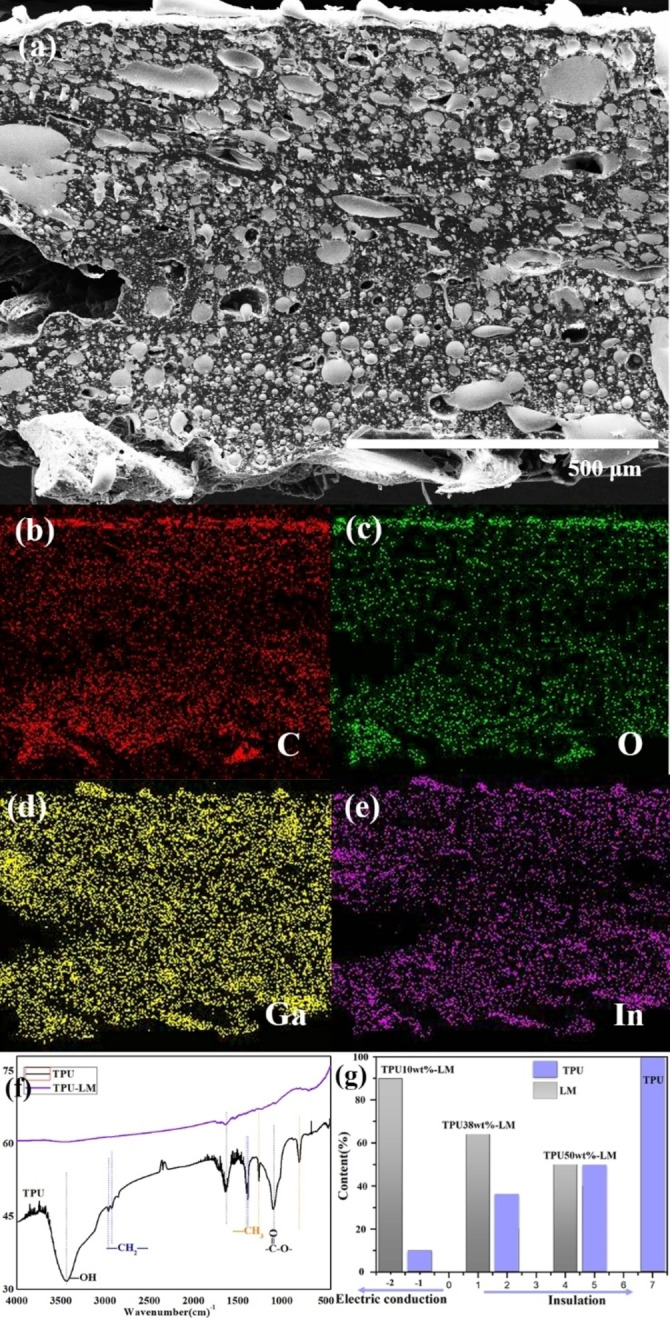
(a) Microstructure of TPU‐LM ink; (b‐e) Energy spectrum of TPU‐LM compound ink; (f) Infrared spectrum comparison between TPU‐LM ink and LM; (g) The relationship between LM addition ratio and conductive or insulation properties in TPU.

SEM and TEM characterized the micromorphology and internal ion valence state of the ink. The structure of TPU‐LM composite material under a scanning microscope is liquid metal particles of 3~5um wrapped by TPU material, as shown in Figure 2a.

The energy spectrum scan of TPU‐LM was obtained by energy spectrum detection, as shown in Figure 2. Red is the C element, green is the O element, yellow is the Ga element, and purple is the In element. TPU is a polymer composite material containing many C and O elements, and the liquid metal is Ga and In blended metal. It could be seen from the figure that the structure of TPU‐LM composite material is a large amount of TPU polymer material wrapped with a small amount of Ga and In elements. According to electron microscope scanning and energy spectrum, the internal structure of TPU‐LM composite material is TPU polymer material wrapped in LM.

The infrared spectrum of liquid metal, TPU polymer, and TPU‐LM composite ink was detected by infrared spectrum, as shown in Figure 2. The 38 wt %TPU‐62 wt %LM ratio composite material does not have electrical conductivity, and it is found to have electrical conductivity after increasing the occupancy ratio of LM by 90 wt%. The electrical conductivity of the TPU‐LM polymer composite is related to the LM and TPU material ratio.

### Preparation of TPU‐LM Micrometer Fiber

**Figure 3:**
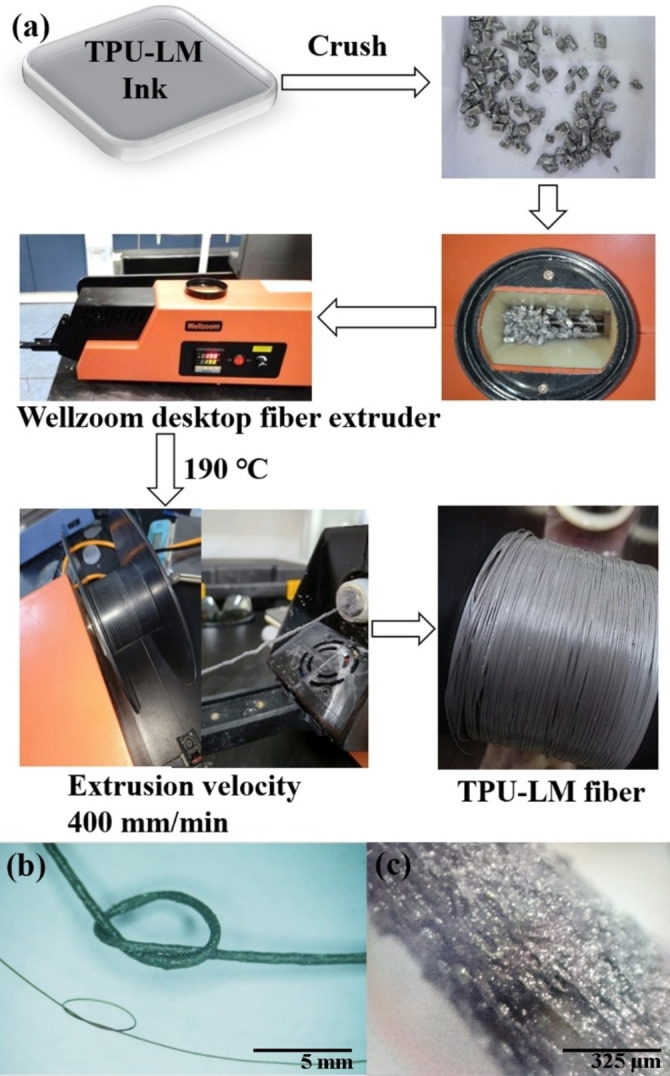
(a) Preparation process of TPU‐LM micron fiber; (b‐c) Microscopic image of TPU‐LM micrometer fiber.

The cured TPU‐LM composite material in the mold was taken out, and it was found that the composite material was in a gray state and had certain flexibility, stretchability, and bendability. Use scissors to cut the material into moderately sized solid small particles, as shown in Figure 3a, to obtain the original particle material of 3D printing consumables.

Turn on the power supply and temperature control switch at the back of the Wellzoom desktop extruder and start working. Press the set key under temperature control to make the number blink and enter the temperature setting state. Set the temperature adjustment at 140 °C and press the Set key again to enter the working state, as shown in Figure 3a. Set the temperature adjustment at 140 °C and press the Set key again to enter the active state, as shown in Figure 3a.

Using the Wellzoom desktop fiber extruder equipment, 600~1750 μm fibers could be prepared at 205 °C, as shown below. Remove the mixture material after the LM and TPU are broken, and place the treated TPU‐LM block particles into the inlet tank of the Wellzoom desktop fiber extruder (Figure 3c). Turn on the main power supply of the Wellzoom desktop fiber extruder and set the extrusion temperature to 140~190 °C. Wait for Wellzoom desktop fiber extruder to heat up and its temperature to stabilize at 190 °C. After 2 to 3 minutes, turn on the working switch of the instrument, adjust the extrusion speed from small to large, adjust the extrusion speed to 400~600 mm/min for wire drawing, wait for the fiber extruder to slowly out, you could continuously get TPU‐LM functional fiber.

Pull out the TPU‐LM wire and connect it to the winding machine for winding, as shown in Figure 3e. TPU‐LM composite fiber was obtained, as shown in Figure 3e. The actual pulled out TPU‐LM printing fiber was shown in Figure 3fg. The composite

fiber was black gray, has certain toughness, hardness is not high. A small segment of a single TPU‐LM functional fiber was measured.

### Characterization of TPU‐LM Printing Fibres

A small segment of a single TPU‐LM functional fiber was measured using a video microscope. Taking the usable fiber with a length of 1.50 mm TPU‐LM, it was found that the diameter of the prepared fiber is 600 μm. It is mainly determined by the drawing caliber of the Wellzoom wire drawing machine, which determines the minimum diameter of the LM‐TPU fiber. The appearance color of the prepared TPU‐LM functional fiber is black with elasticity, as shown in figure 3. Since the diameter of the fiber is in the micrometer scale, the three images in Figure 3gef show the elastic black fiber filament taken under a video microscope.

Test the TPU50 wt%‐LM functional fiber resistance, which has insulation, and the LM‐TPU composite is insulated at a 50 % ratio. The LM is conductive but exists mainly as tiny droplets in the insulating TPU. Its insulation may be because the 50 wt% TPU polymer wraps the LM droplets and does not form a small conductive path inside the fiber, resulting in no internal conduction. The TPU50 wt%‐LM fiber was not conductive inside; the resistance was infinite, so it could not form a current path, and it could not release heat to the outside.

The liquid metal fiber is tightly wound with a tension meter, and the elongation ratio of the fiber is recorded. When the liquid metal fiber was continuously stretched, the tension and elongation displacement of the liquid metal fiber could be obtained. The liquid metal fibers were found to break at 0.72 N tension. At this time, the total elongation of the liquid metal fiber was 48.10 mm. The diameter of TPU‐LM fiber is 600 μm, and the cross‐sectional area of the TPU‐LM microfiber is 1.1304 mm^2^. Therefore, according to the stretching limit formula: 
(1)

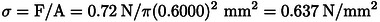




σ is the tensile limit, F is the tensile force, and A is the cross‐sectional area. Therefore, the elastic and tensile limits of the functional fiber are 0.637 N/mm^2^.

### 3D Printing Flexible Electronic Materials

**Figure 4:**
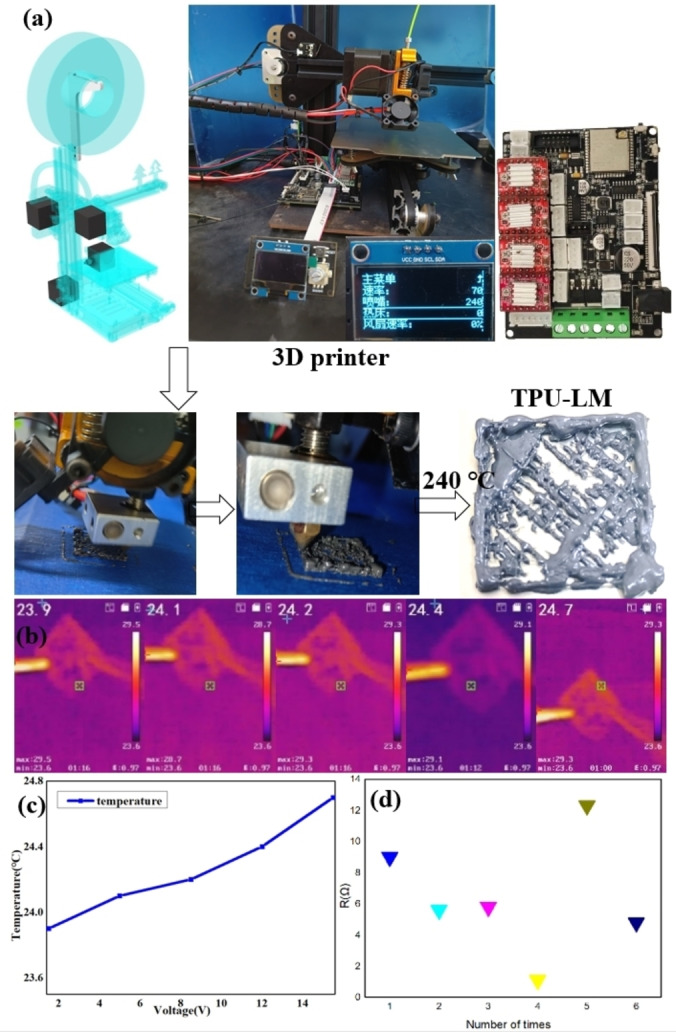
(a) 3D printer printing process; (b–c) the temperature of the printing device increases with the increase of voltage; (d) Resistance variation of printing devices.

Purchase the new 3D printer (Sapling T4) and start printing TPU‐LM consumables after repeated testing. The purchased Sapling T4 3D printer would be assembled according to the installation instructions, and the components will be pre‐processed. The film is torn on the acrylic profile; the sticker is opened on the metal bottom plate, the thermistor and nozzle are reinforced, the motherboard is installed and driven, dispose of the excess of the limit switch. Assemble the idler wheel, limit switch, motor, pulley, and other minor modules of the 3D printer. The metal base plate is connected to the YZ profile, the whole installation is performed, and the XYZ shaft motor, block, screw, and idler are installed. Install the driver board and adjust the drive voltage to 0.5 V and current to 0.6 A, as shown in Figure 4a. Connect the electrical route according to the circuit diagram, as shown in Figure 4b, connect the screen, and sort out the electrical route. Connect the power supply to start, check whether the nozzle and hot bed temperature are normal temperature, and check whether the average fan and controllable fan work normally. Adjust the rotation direction of the XYZ axis and the extruder machine, and then zero the XYZ axis and adjust the limit switch of the axis so that the interval between the nozzle and the print panel is exactly the thickness of a piece of A4 paper. The SD card of the downloaded model is inserted into the 3D printer for printing. The side length is 2 cm, and the shape is a complete cube. The 3D printer could complete the printing work typically.

Debug the 3D printer (Figure 4a) and preheat it to 190 °C. Import the required print model and adjust the model print parameters. Connect the computer to download the model and print the model as a 2 cm square grid. Set the printing temperature to 240 °C, the printing rate to 70 %, the hotbed temperature to 0 (normal temperature), and the controlled fan rate to 0 %.

The effect of 3D printing of TPU‐LM composite material on the PEI panel is shown in Figure 4. Connect the printed model to the electrical appliance. Take 1.5 V voltage as the initial voltage and increase the voltage by 3.5 V each time. After 10 min, test the current temperature with a visual thermometer, and obtain the data as shown in figure 4(b) below. As can be seen from figure 4(b‐c), the temperature of the model prepared by 3D printing gradually increases with the voltage increase.

Cut the printed 3D model and connect the multimeter to measure the slope resistance at different positions. It was found that the slope resistance of different positions is additional. It could be seen that conductive 3D printed electronic devices, which have conductivity, heat dissipation, elasticity, and tensile properties have been prepared. When the proportion of TPU in LM‐TPU composite consumables is too high, the printed circuit has poor electrical conductivity.

After 3D printing electronic devices using LM‐TPU, the devices could be stored for a long time at room temperature without being destroyed. This is because the TPU polymers have an excellent ability to protect LM droplets. At the same time, the melting temperature of TPU is also higher than 190–230 °C. Therefore, the use of TPU‐LM ink printing devices, in general, can be stored for a longer time. However, because the melting point of LM‐TPU ink is 149.8 °C, therefore, when the temperature is too high, it may cause damage to the electronic device. LM‐TPU printed electronics were not resistant to high temperatures and open flames. At the same time, when encountering a strong acid or a strong base, a chemical reaction would occur with the polymer material. Therefore, LM‐TPU printed electronics were also not resistant to strong acids and bases.

## Conclusions

Through systematic experimental research in this paper, the following main conclusions could be drawn: TPU‐LM composite inks with different TPU ratios (50–10 wt%) were prepared. The melting point of TPU‐LM was 149.8 °C, which had firm adhesion and could be adhered to the glass surface. This ink has good flexibility, stretchability, and bendability. A large amount of TPU polymer was coated with a small amount of LM(EGaIn) in this composite ink. Using the Wellzoom desktop extruder, the mixed ink is prepared as a black and elastic functional fiber with a diameter of 600 um. The size, electrical conductivity, heat dissipation and tensile properties of these fibers were tested. This fiber has excellent elasticity, toughness, hardness is not high, fracture at 0.72 N tension, and tensile limit is 0.637 N/ mm^2^. Through 3D printing, the TPU‐LM fiber was printed at a printing temperature of 240 °C. The slope of the TPU‐LM composite material is electrically conductive, and its resistance decreases with the increase of external pressure.

## Experimental Method

The metal gallium (Ga) and metal indium (In) were fused under the condition of 60 °C in an appropriate proportion, and the LM alloy was formed by mixing and stirring for 30 minutes. Thermoplastic polyurethane elastic TPU({[CONHRNHCOOR′O]‐[CONHRNHCOO~O]_m_}_n_) was purchased from Mooz Co., LTD.

Sapling T4 3D printer was purchased from Sapling Technology Co., LTD. The’ Print size of 3D printer is 120*120*160 mm; the printing accuracy is 0.015~0.05 mm; the printing speed is 40~60 mm/s; and the operating voltage is 12 V and the power is 55 w.

The preparation of TPU‐LM ink: 50 wt% LM and small particles of 50wt %TPU are mixed in a beaker and heated at 190 °C to melt into a molten state. Continuous stirring makes the two substances fully mixed to form a uniform black mixed ink. This mixed ink is viscous and has poor fluidity. This mixed ink was poured into the mold while it is hot, and the solid TPU‐LM composite material was obtained after cooling.

Three‐dimensional (3D) printing: Set the 3D printer operating zero. Turn on the machine power until the screen enters the home page, click the module motion control interface button to enter the interface. Click the module reset button until the machine initialization action was complete. Click the zero‐setting button to record the zero position, and the Z‐axis would be set to “0”. Click the module reset button to keep the nozzle away from the hot bed. Click the screen module icon to enter the interface, and click the nozzle heating button to make the temperature reach 200 °C. Straighten the consumable fiber manually, insert the consumable through the upper hole, and press down firmly. Click the button until supplies flow out of the nozzle.

## Supporting Information

The authors have cited additional references within the Supporting Information.

## Conflict of Interests

The authors declare no conflict of interest.

1

## Supporting information

As a service to our authors and readers, this journal provides supporting information supplied by the authors. Such materials are peer reviewed and may be re‐organized for online delivery, but are not copy‐edited or typeset. Technical support issues arising from supporting information (other than missing files) should be addressed to the authors.

Supporting Information

## Data Availability

The data that support the findings of this study are available from the corresponding author upon reasonable request.

## References

[1] H. Aaron , T. Ravi , M. S. Gwyneth , D. B. Michael , J. M. Eric , Adv. Mater. 2022, 34, 2200182.

[2] Q. Wang , X. Y. Ji , X. Liu , Y. Liu , J. J. Liang , ACS Nano 2022, 16, 12677–12685. 35926219 10.1021/acsnano.2c04299 PMC9413406

[3] S. M. Moon , H. U. Kim , K. Lee , J. W. Park , Y. H. Kim , S. Q. Choi , iScience 2021, 24, 103183. 34703989 10.1016/j.isci.2021.103183 PMC8524151

[4] S. T. Liang , J. Yang , F. J. Li , S. B. Xie , N. Song , L. Hu , RSC Adv. 2023,13, 26650–26662. 37681047 10.1039/d3ra04356h PMC10481125

[5] S. T. Liang , F. J. Li , S. B. Xie , Y. Hu , X. Qu , L. Zhou , Mater. Today Commun. 2023, 36, 106894.

[6] S. T. Liang , J. Li , F. J. Li , L. Hu , W. Chen , C. Yang , ACS Omega 2022, 7(15), 12891–12899. 35474773 10.1021/acsomega.2c00098 PMC9025990

[7] D. B. Michael , K. Navid , J. P. Matthew , M. Carmel , P. Natl. Acad. Sci. USA 2017, 114, 2143–2148.

[8] W. L. Geon , P. Min , Y. K. Junk , Composites Part A 2005, 37, 5.

[9] M. G. Mohammed , R. Kramer , Adv. Mater. 2017, 29, 19. 10.1002/adma.201604965 28247998

[10] C. I. Ade , A. F. Chrimes , A. Zavabeti , K. J. Berean , B. J. Carey , J. C. Zhuang , Y. Du , S. X. Dou , K. Suzuki , R. A. Shanks , R. Nixon-Luke , G. Bryant , K. Khoshmanesh , K. Kalantar Zadeh , T. Daeneke , Nano Lett. 2017, 17, 12. 10.1021/acs.nanolett.7b04050 29095626

[11] Y. L. Lin , C. Cooper Small 2015, 11, 48. 10.1002/smll.201502692 26568095

[12] S. H. Jeong , S. Chen Sci. Rep. 2015, 5, 18257. 26671673 10.1038/srep18257 PMC4680911

[13] D. Uranbileg , D. Y. Osman , Adv. Mater. Technol. 2018, 3, 4.

[14] M. D. Bartlett , A. Fassler , Adv. Mater. 2016, 28, 19. 10.1002/adma.201670133 27167031

[15] H. Z. Wang , Y. Y. Yao , X. J. Wang , ACS Omega 2019, 4, 2311–2319. 31459473 10.1021/acsomega.8b03466 PMC6648024

[16] T. Ravi , K. Steven , ACS Appl. Mater. Inter. 2019, 11, 17873–17883. 10.1021/acsami.9b04569 31007016

[17] E. J. Markvicka , M. D. Bartlett , Nat. Mater. 2018, 17, 618–624. 29784995 10.1038/s41563-018-0084-7

[18] S. Park , G. Thangavel , Adv. Mater. 2019, 31, 1. 10.1002/adma.201805536 30387213

[19] H. Z. Wang , Y. Y. Yao , Adv. Mater. 2019, 31, 1901337.

[20] D. D. Michael , Adv. Mater. 2017, 29, 27.

[21] S. H. Jeong , ACS Appl. Mater. Interfaces 2017, 9, 18. 10.1021/acsami.7b04752 28453282

[22] Z. Mingkuan , Z. Pengju , Appl. Mater. Today 2020, 19, 100612.

[23] P. Minwoo , I. Jungkyun , S. Minkwan , M. Yuho , P. Jaeyoon , C. Heesook , P. Soojin , S. M. Bo , J. Sanghun , C. D. Young , B. Jihyun , P. Jongjin , J. Unyong , K. Kinam , Nat. Nanotechnol. 2012, 7, 12.

[24] Y. L. Lin , C. Cooper , Small 2015, 11, 48. 10.1002/smll.201502692 26568095

[25] B. Michael , D. F. Andrew , K. Navid , Ma. Eric , J. M. Pratiti , M. J. Carmel , Adv. Mater. 2016, 28, 19.

[26] H. Z. Wang , Y. Y. Yao , X. J. Wang , L. Sheng , X. H. Yang , Y. T. Cui , P. J. Zhang , W. Rao , R. Guo , S. T. Liang , W. W. Wu , J. Liu , Z. Z. He , ACS Omega 2019, 4, 1. 31459473 10.1021/acsomega.8b03466 PMC6648024

[27] S. H. Jeong , S. Chen , J. X. Huo , E. K. Gamstedt , J. H. Liu , S. L. Zhang , Z. B. Zhang , K. Hjort , Z. G. Wu , Sci. Rep. 2015, 5. 10.1038/srep18257 PMC4680911 26671673

[28] W. Phillip , S. V. Connor , Z. Mason , Cheng feng Pan , V. Michael , K. P. Dinesh , H. K. Seung , L. M. Walker , M. J. Carmel , ACS Appl. Mater. Interfaces 2022, 14(49), 55028–55038. 36458663 10.1021/acsami.2c14815

[29] K. Minwoo , L. Hyungjun , H. K. Seung , Adv. Sci. 2023, 10, 2205795.

[30] K. Minwoo , C. Chulmin , S. Wooseop , J. P. Jung , K. Jaewon , W. Phillip , M. J. Carmel , H. K. Seung , NPJ Flex. Electron. 2022, 6, 99.

[31] C. Chulmin , S. Wooseop , K. Minwoo , B. Junhyuk , W. Phillip , H. Sukjoon , H. K. Seung , Small 2022, 18, 2202841.

[32] G. Z. Chen , H. M. Wang , ACS Appl. Mater. Interfaces 2020, 12, 5. 10.1021/acsami.9b17659 31939285

[33] S. Nagels , W. Deferme , Materials 2018, 11, 3. 10.3390/ma11030375 PMC5872954 29510497

[34] B. O. Kadri , Adv. Mater. Interfaces 2018, 5, 10.

[35] L. Joshua , A. Stephen , Adv. Funct. Mater. 2015, 25, 9.

[36] Y. L. Lin , C. Cooper , Small 2015, 11, 48. 10.1002/smll.201502692 26568095

[37] G. S. Maricruz , S. X. Cai , Small 2020, 16, 12.

[38] Y. G. Park , H. Min , Nano Lett. 2019, 19, 8. 30983359 10.1021/acs.nanolett.9b00150

