# Supplementary material for: 3D Printing of TPU‐Liquid Metal Composite Inks for the Preparation of Flexible Sensing Electronics
Source: ChemistryOpen. 2024 Apr 26;13(9):e202300301. doi: 10.1002/open.202300301 (PMC11467734; doi:10.1002/open.202300301)
Supplement: Supplementary file 1 — Supporting Information [file OPEN-13-e202300301-s001.pdf]

ChemistryOpen

Supporting Information

3D Printing of TPU-Liquid Metal Composite Inks for the Preparation of Flexible Sensing Electronics

Shuting Liang,* Mengjun Huang, Dabo Jiang, Jianyang Chen, Liang Hu, Jiujia Chen, and Zhezi Wang

Supplemental materials

Figure S1. (a-b) Fiber extrusion diagram; (c) Wellzoom desktop extruder front view; (d) Wellzoom desktop extruder side view; (e) the discharge effect of extruded fiber; (f) the discharge of extruded fibers; (g) the temperature of the extruded fiber.

Figure S2. The size of liquid metal droplets in TPU ink is about 2~14 μm.

Figure S3. (a) Fibers without tension; (b) the fibers after pulling.

Figure S4. Small particle of TPU-LM composites solid.

Figure S5. (a) 3D printer of MOOZ that could not print TPU-LM; (b) Small tree T4-3D printer that could print TPU-LM; (c) Print software control interface.

Figure S6. Profile resistance of TPU-LM composites: (a) Profile resistance at position A; (b) Profile resistance at position B.

Figure S7. The resistance of LM-TPU model decreases with the increase of pressure. When the pressure reaches a certain level, the model resistance does not change.

Figure S8. (a) Copper wire as inner core LM-TPU functional fiber; (b) Thermal image of Cu@ TPU-LM functional fiber with different voltage applied to both ends; (c) Thermal image of pure Cu wire with different voltages at both ends.

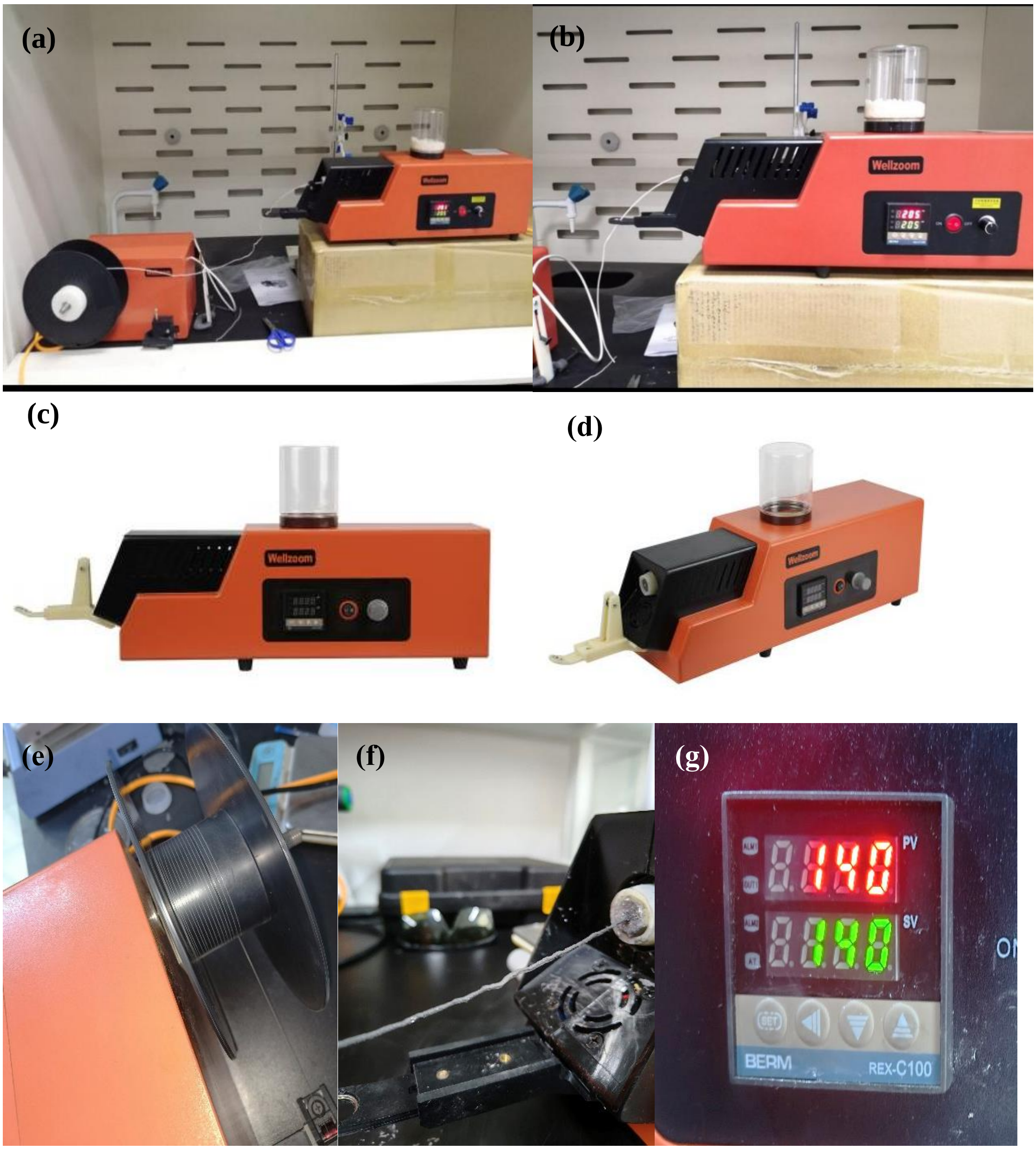

Figure S1. (a-b) Fiber extrusion diagram; (c) wellzoom desktop extruder front view; (d) wellzoom desktop extruder side view; (e) the discharge effect of extruded fiber; (f) the discharge of extruded fibers; (g) the temperature of the extruded fiber.

Wellzoom desktop extruder equipment parameters:

(1) Extrusion speed: 300~650 mm/min;

(2) Extrusion diameter: 1.75mm & 3.00mm;

(3) extrusion line diameter tolerance: +/- 0.05mm (1.75mm); +/- 0.1mm (3.00mm).

(4) Equipment size: 560 mm×160 mm×200 mm.

(5) Operating temperature: room temperature to below 300 °C.

(6) Power: 120W;

(7) Power supply: 220 V, 50 Hz.

Use process of the instrument:

Turn on the backup power switch of the equipment and let the temperature control start to work. Set the temperature, wait for the temperature to rise to the set temperature, and let the temperature balance. Pour the sample into the hopper and add the particle sample in time. Press the motor switch to start the motor. After the fiber was extruded, pay attention to whether the two fulcrum points of the support were in the appropriate position, and adjust the position of the front and back of the support to ensure the smooth production of the fiber. Adjust the speed counterclockwise to accelerate and clockwise to slow down. After fiber processing, turn off the motor and turn off the power.

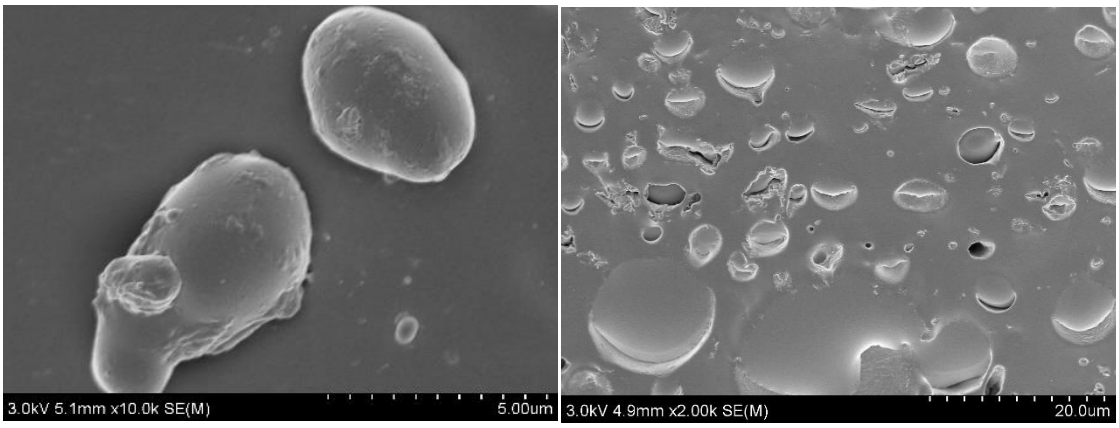

Figure S2. The size of liquid metal droplets in TPU ink is about 2~14 μm.

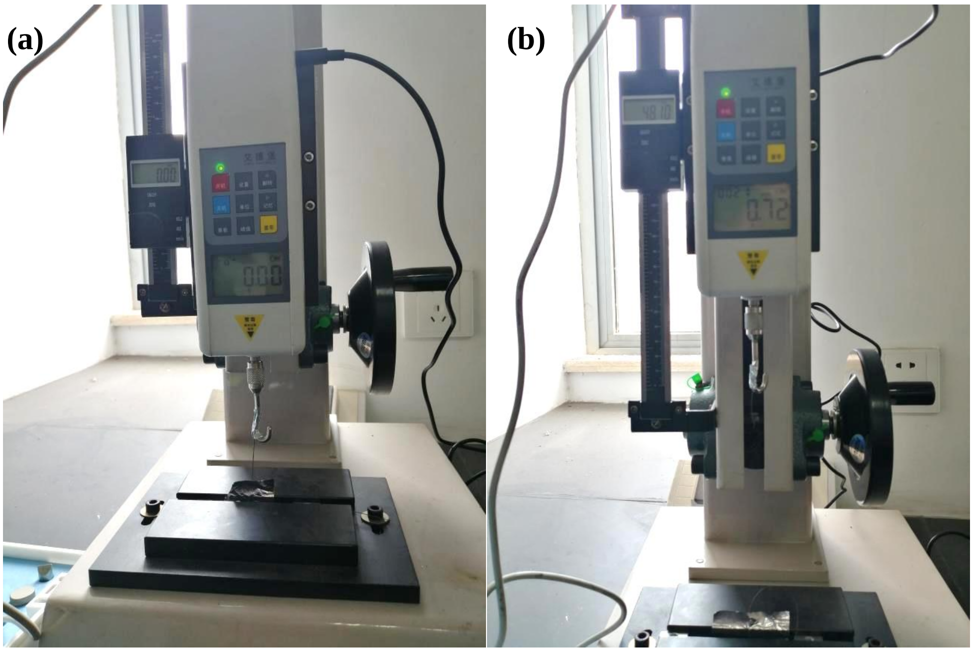

Figure S3. (a) Fibers without tension; (b) the fibers after pulling.

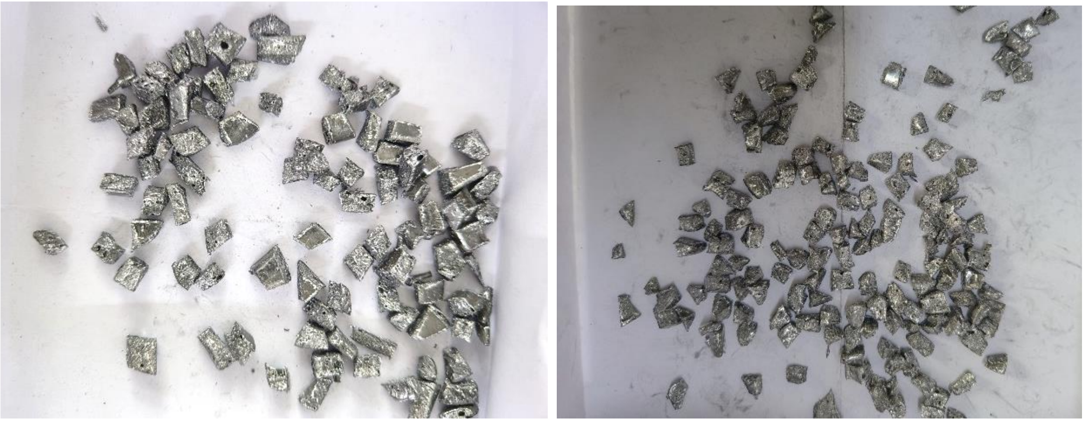

Figure S4. Small particle of TPU-LM composites solid.

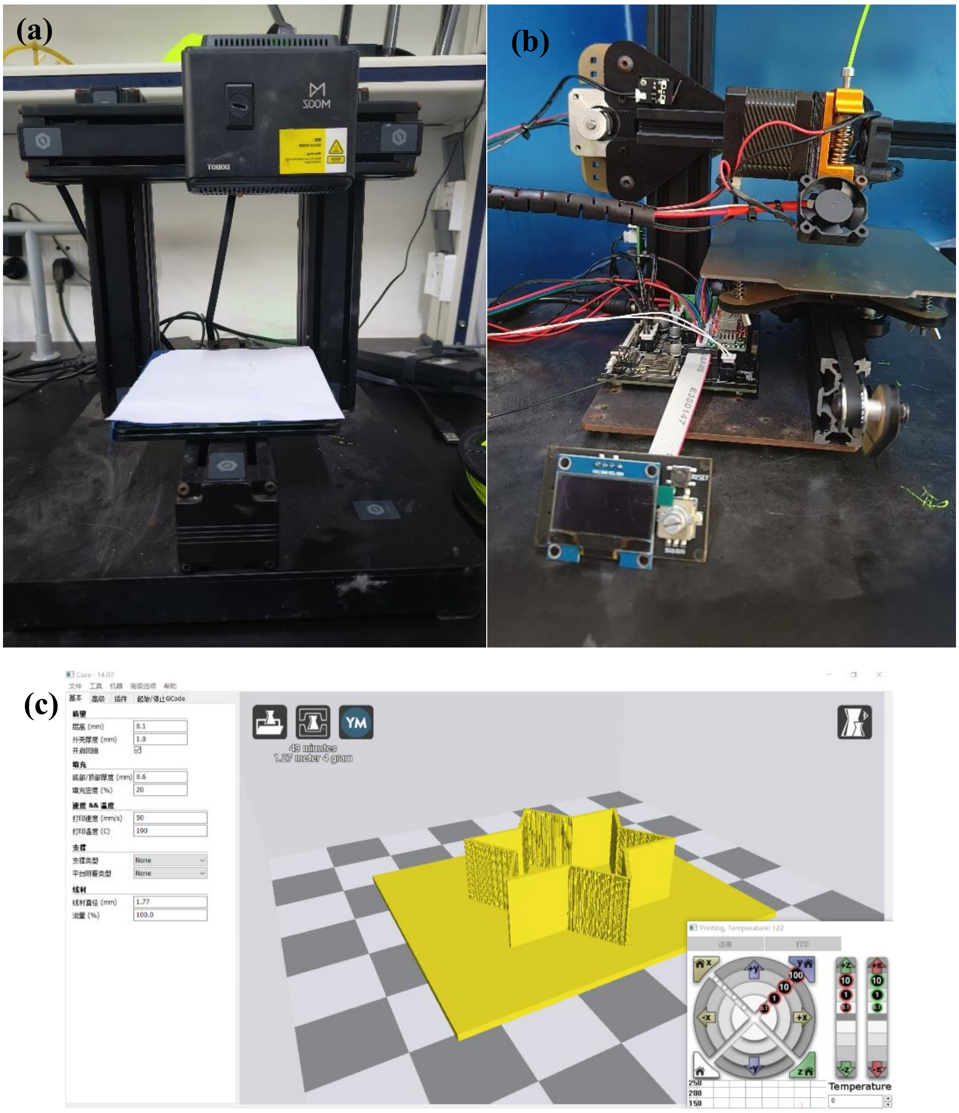

Figure S5. (a) 3D printer of Mooz that could not print TPU-LM; (b) Small tree T4-3D printer that could print TPU-LM; (c) Print software control interface.

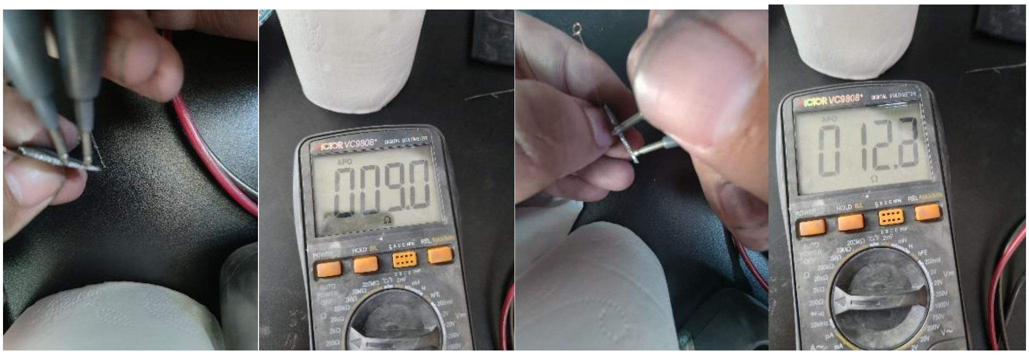

Figure S6. Profile resistance of TPU-LM composites: (a) Profile resistance at position A; (b) Profile resistance at position B.

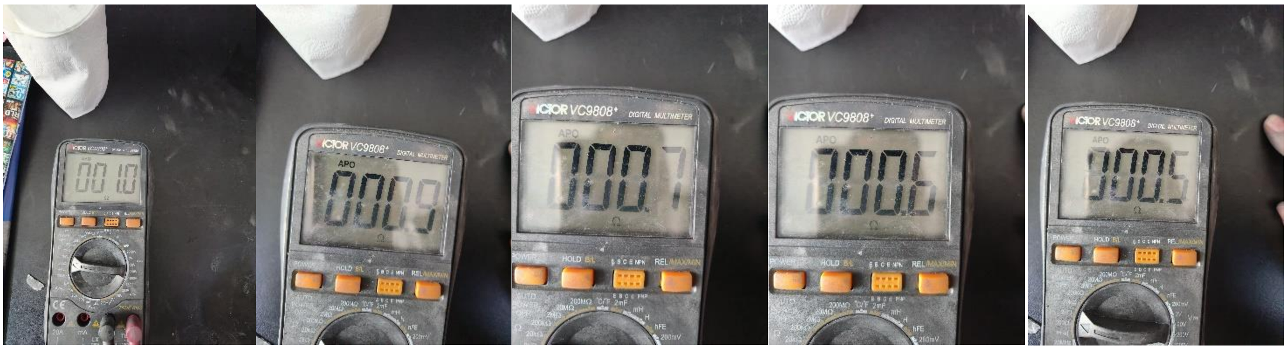

Figure S7. The resistance of LM-TPU model decreases with the increase of pressure. When the pressure reaches a certain level, the model resistance does not change.

Preparation process of Cu@LM-TPU fiber

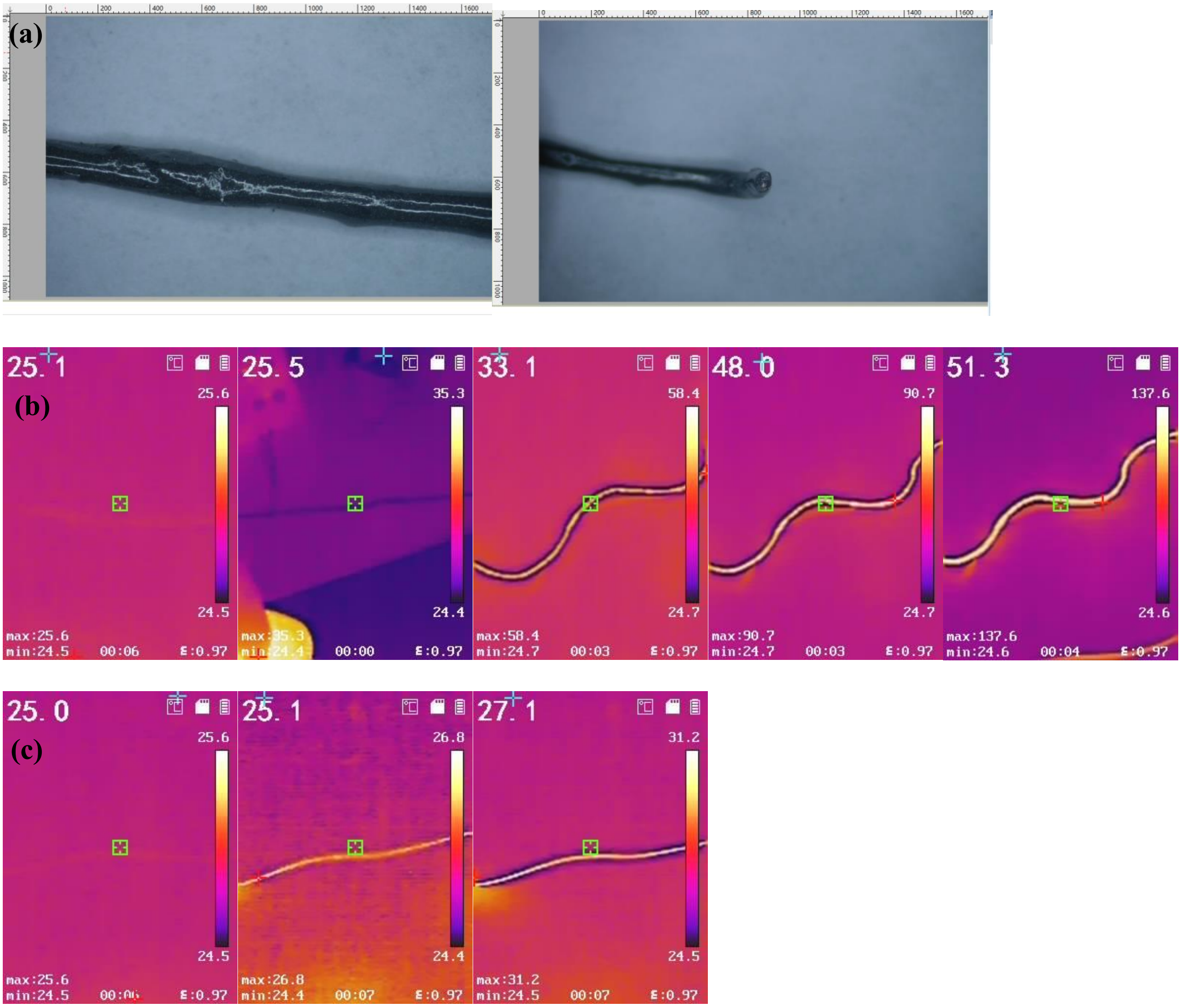

Figure S8. (a) Copper wire as inner core LM-TPU functional fiber; (b) Thermal image of Cu@ TPU-LM functional fiber with different voltage applied to both ends; (c) Thermal image of pure Cu wire with different voltages at both ends.

The copper wire was wrapped with TPU-LM as the shell to form Cu@ TPU-LM fiber, which could enhance its electrical conductivity. A voltage of 0, 1, 2, 3, 4 V was applied to both ends of the functional fiber, and its thermal imaging is shown in Figure S7 (b). The Cu@ TPU-LM after the voltage was applied at both ends of the functional fiber, the voltage from left to right is 0, 1, 2, 3, 4 V respectively, and the corresponding heating temperature is 25.1 °C, 25.5 °C, 33.1 °C, 48.0 °C, 51.3 °C. With the increase of

voltage at both ends of the fiber, the heat released by the fiber continues to increase, and the temperature rise changes very rapidly, and no short circuit occurs. The thermal image of the same copper wire with a voltage of 0, 1, 2 V is shown in the figure S7 (c). The voltage 0, 1, 2 V is applied to both ends of the pure copper wire, and the corresponding temperature is 25.0 ℃, 25.1 ℃, 27.1 ℃, and the thermal change amplitude is not obvious after the voltage is added.

When the voltage of pure copper wire was too large, the thermal change was not obvious and the temperature change is not large. The higher the voltage on Cu@ TPU-LM functional fiber, the more obvious the heat release effect, and the more obvious the temperature change range. The melting point of this Cu@ TPU-LM fiber was 180 ℃.
